# Uneven Ground: Survival Differences Among Victorian Lung Cancer Patients by Location of Residence (2011–2023): A Retrospective Cohort Study

**DOI:** 10.5694/mja2.70268

**Published:** 2026-08-18

**Authors:** Evangeline Samuel, Eldho Paul, Mike Lloyd, Sanuki Tissera, Craig Underhill, Sagun Parakh, Phillip Parente, Inger Olesen, Javier Torres, Katharine See, Gavin M. Wright, David Langton, Thomas John, Matthew Conron, James Bartlett, Nicola Atkin, Nikolajs Zeps, Susan V. Harden, Wasek Faisal, John R. Zalcberg, Rob G. Stirling

**Affiliations:** ^1^ School of Public Health and Preventive Medicine Monash University Melbourne Victoria Australia; ^2^ Department of Medical Oncology Latrobe Health Services Traralgon Victoria Australia; ^3^ Department of Medical Oncology Alfred Health Melbourne Victoria Australia; ^4^ Border Medical Oncology Research Unit Albury Wodonga Regional Cancer Centre Albury New South Wales Australia; ^5^ Rural Medical School Albury UNSW Sydney Albury New South Wales Australia; ^6^ Olivia Newton‐John Cancer Wellness and Research Centre Melbourne Victoria Australia; ^7^ Department of Medical Oncology Eastern Health Melbourne Victoria Australia; ^8^ Andrew Love Cancer Centre Barwon Health Geelong Victoria Australia; ^9^ Department of Medical Oncology Goulburn Valley Health Shepparton Victoria Australia; ^10^ Department of Respiratory Medicine Northern Health Melbourne Victoria Australia; ^11^ Department of Cardiothoracic Surgery University of Melbourne Melbourne Victoria Australia; ^12^ Department of Surgery St Vincent's Hospital Melbourne Melbourne Victoria Australia; ^13^ Department of Thoracic Medicine Peninsula Health Melbourne Victoria Australia; ^14^ Department of Medical Oncology Victorian Comprehensive Cancer Centre Melbourne Victoria Australia; ^15^ Faculty of Medicine, Dentistry and Health Sciences University of Melbourne Melbourne Victoria Australia; ^16^ Peter Mac Callum Cancer Centre Melbourne Victoria Australia; ^17^ Department of Respiratory Medicine St Vincent's Hospital Melbourne Melbourne Victoria Australia; ^18^ Department of Respiratory Medicine Western Health Melbourne Victoria Australia; ^19^ Parkville Integrated Palliative Care Service Peter MacCallum Cancer Centre Melbourne Victoria Australia; ^20^ Central Clinical School, Faculty of Medicine, Nursing and Health Sciences Monash University Melbourne Victoria Australia; ^21^ Department of Radiation Oncology Peter Mac Callum Cancer Centre Melbourne Victoria Australia; ^22^ Department of Medical Oncology Grampians Health Ballarat Victoria Australia; ^23^ Department of Respiratory Medicine Alfred Health Melbourne Victoria Australia; ^24^ School of Translational Medicine Monash University Melbourne Victoria Australia

**Keywords:** cancer, lung diseases, registries, rural health services, survival analysis

## Abstract

**Objectives:**

Patients in regional and rural areas consistently experience poorer lung cancer survival rates compared with those in metropolitan centres, but the reasons remain unclear. This study examined survival differences in non‐small cell lung cancer (NSCLC) across Victoria and identified key prognostic factors contributing to these differences.

**Design:**

Retrospective cohort study.

**Setting and Participants:**

NSCLC patients diagnosed between 1 July 2011 and 22 May 2023 identified from the Victorian Lung Cancer Registry (VLCR).

**Main Outcome Measures:**

Residential address and treatment institution were classified using the Modified Monash Model (MMM): Modified Monash (MM) category 1 (MM1) as metropolitan, MM2 as regional and MM3–MM7 as rural/remote. Demographic, socio‐economic and cancer‐specific factors were analysed as potential predictors of all‐cause mortality.

**Results:**

Among 13,548 patients, 4244 (31%) lived in regional or rural/remote areas. Compared with metropolitan patients, these groups had higher smoking prevalence (metropolitan, 2848/9304 [31%] vs. regional, 366/1083 [34%] vs. rural, 1148/3161 [37%]) and were more likely to be Australian‐born (metropolitan, 4919/9304 [53%] vs. regional, 873/1083 [81%] vs. rural, 2603/3161 [82%]; *p* < 0.001). Comorbidity burden was similar across groups (median, 1; interquartile range, 0.0–1.0; *p* = 0.19). Socio‐economic disadvantage was more marked in regional and rural patients (median Index of Relative Socio‐Economic Advantage and Disadvantage [IRSAD] deciles: metropolitan, 8.0 vs. regional, 5.0 vs. rural, 3.0; *p* < 0.001), and average travel times to treatment were longer (metropolitan, 0.4 vs. regional, 1.9 vs. rural, 2.8 h, respectively). Patients treated at regional institutions had poorer survival (hazard ratio [HR], 1.27; 95% confidence interval [CI], 1.19–1.35; *p* < 0.001). This difference persisted after adjustment for age, stage, performance status, smoking and comorbidities (HR, 1.11; 95% CI, 1.04–1.18; *p* = 0.001).

**Conclusions:**

Regional, rural and remote patients with NSCLC face greater socio‐economic disadvantage and travel burdens, and experience poorer survival even after accounting for clinical and demographic factors. These findings highlight enduring inequities in lung cancer care and emphasise the need for targeted interventions to strengthen access, treatment equity and outcomes for non‐metropolitan populations.

## Plain Language Summary

**The known:** Lung cancer is the leading cause of cancer mortality in Australia. Regional, rural and remote patients have consistently worse survival rates. The reasons for poorer survival are not fully understood.

**The new:** Using Victorian Lung Cancer Registry data, we observed that rural patients face longer travel times, socio‐economic disadvantage and poorer survival when treated at regional institutions.

**The implications:** Survival disparities are not fully explained by clinical or demographic factors, suggesting structural healthcare inequities. Improving timely diagnosis, treatment, multidisciplinary care and supportive services is critical. Findings inform policy, service provision and lung cancer screening programs to ensure equity for rural populations.

## 1 Introduction

Lung cancer is the fifth most commonly diagnosed cancer in Australia and the leading cause of cancer‐related mortality worldwide [1, 2, 3]. In Australia, lung cancer incidence has risen over the past decade, with a higher burden in regional and remote areas [3]. Between 2012 and 2016, age‐standardised incidence rates for lung cancer were 21% higher in remote and very remote areas compared with major cities [3].

This geographic difference extends to mortality across all Australian cancers. From 2001 to 2010, there were an estimated 8878 excess cancer deaths in regional and rural areas (95% confidence interval [CI], 8187–9572) compared with metropolitan cities [4]. Among men, the age‐standardised mortality ratio comparing regional and remote areas with metropolitan areas showed no improvement over time, increasing from 1.08 in 1997–2000 to 1.11 in 2006–2010. Among women, the disparity also increased, with the corresponding mortality ratio rising from 1.01 to 1.07 over the same period [4]. Lung cancer follows a similar pattern: between 2015 and 2019, the mortality rates were 41 deaths per 100,000 people in very remote areas compared with 27 deaths per 100,000 people in major cities [5].

Data for the state of Victoria mirror these national trends. Between 2018 and 2022, the 5‐year survival rate for patients with lung cancer living outside major cities was 7% lower than for those in metropolitan areas (25% vs. 32%) [6]. Despite substantial advances in lung cancer treatments, including surgery, chemotherapy, immunotherapy and targeted therapies, access to these treatments remains challenging for regional populations. Barriers such as prolonged travel requirements, socio‐economic disadvantage, limited referral and access to specialist care, delayed diagnosis and reduced participation in clinical trials may contribute to poorer outcomes [7].

However, although these survival disparities are well documented, the underlying reasons remain poorly understood. Existing reports largely rely on aggregated or administrative data and rarely incorporate patient‐level clinical, treatment or health service variables. Few studies differentiate between residence and treatment location. Moreover, little is known about which prognostic factors are most relevant and potentially modifiable in regional settings.

We aimed to address these critical gaps by analysing detailed, population‐level data from the Victorian Lung Cancer Registry (VLCR; https://vlcr.org.au/) to explore both survival differences and the underlying drivers of those differences across geographic settings in Victoria. Specifically, this study aimed to identify the key prognostic factors contributing to disparities in non‐small cell lung cancer (NSCLC) outcomes using a state‐wide, patient‐level data from the VLCR [8] and to examine survival differences in NSCLC between metropolitan and regional, rural and remote populations in Victoria.

## 2 Methods

### 2.1 Study Design and Setting

We conducted a population‐based retrospective cohort study using VLCR data from 19 health systems and 50 hospitals. This study is reported in accordance with the STROBE guidelines (Table S1).

### 2.2 Study Participants

We included all newly diagnosed NSCLC cases in the VLCR between 1 July 2011 and 22 May 2023, identified using International Classification of Diseases‐10 (ICD‐10) codes (C34.0–C34.9, Z85.2). Patients with mesothelioma, small cell lung cancer and non‐primary site of malignancy were excluded.

### 2.3 Data Variables and Sources

Data collected included date of birth, sex, year of diagnosis, smoking status, continent of birth, comorbidities, indigenous status and performance status at diagnosis using the Eastern Cooperative Oncology Group (ECOG) scale [9]. Sex was recorded in the registry as male or female at the time of diagnosis. Information on gender identity was not collected; therefore, analyses and reporting refer to sex rather than gender. Cancer‐related characteristics included clinical stage (International Association for the Study of Lung Cancer [IASLC], Tumour Node Metastasis [TNM] 8th edition) [10], histology, treatment received and clinical trial enrolment. Comorbidity data included diabetes mellitus (type 1 and 2), renal insufficiency (dialysis or serum creatinine > 300 μmol/L), cardiovascular disease (history of myocardial infarction or coronary intervention), respiratory disease (forced expiratory volume [FEV_1_] < 66%) and other cancer diagnoses (excluding cutaneous basal or squamous cell carcinoma). Dates of death were obtained from the Victorian Registry of Births, Deaths and Marriages (https://www.bdm.vic.gov.au/).

Residence at cancer diagnosis was collected through several sources in the VCLR. Patient location of residence at cancer diagnosis was classified as metropolitan, rural, remote or very remote based on the Modified Monash Model (MMM) [11]. The model measures remoteness and population size on a scale of Modified Monash (MM) category 1–7 (MM1 represents major cities and MM7 very remote areas). The treatment institution, where patients received systemic anti‐cancer therapy, was also classified according to the MMM. Geographic location was presented descriptively as three categories (MM1 metropolitan, MM2 regional and MM3–MM7 rural and remote); however, for survival analyses, regional and rural/remote categories were combined to form a single non‐metropolitan group (MM2–MM7). Systemic anti‐cancer therapy encompassed chemotherapy, immunotherapy and targeted therapy. The driving distance was calculated as the distance between the patient's residential postcode and the systemic anti‐cancer therapy institution [12].

The Socio‐Economic Indexes for Areas (SEIFA) were matched to patient postcodes, obtained from the SEIFA website and linked to our dataset [13]. SEIFA provides the Index of Relative Socio‐Economic Advantage and Disadvantage (IRSAD), combining information on income, education and employment to give a general picture of an area's socio‐economic status within geographic areas. IRSAD is ranked from decile 1 (most disadvantaged) to decile 10 (most advantaged) [14].

### 2.4 Statistical Analyses

Patient and disease characteristics were compared between metropolitan and regional, rural and remote groups using χ^2^ tests for categorical variables, and Student's *t*‐test or Wilcoxon rank‐sum tests for normally and non‐normally distributed continuous variables, respectively. Overall survival was defined from the date of diagnosis to death or the census date (22 May 2023). Kaplan–Meier curves were used to estimate survival, with differences assessed using the log‐rank test.

Univariable and multivariable Cox proportional hazards regression analyses were performed to identify factors associated with overall survival. Variables with *p* < 0.05 in univariable analysis or considered clinically relevant were included in a hierarchical multivariable model to assess the independent impact of treatment institution location. Results are presented as hazard ratios (HRs) with 95% confidence intervals (CIs).

Propensity scores, representing the probability of group assignment conditional on observed baseline covariates, were derived from age, sex, ECOG performance status, smoking status, clinical stage, number of comorbidities and year of diagnosis and were applied for matching to achieve covariate balance between comparison groups. This propensity score–matched analysis was conducted as a sensitivity analysis.

All statistical tests were two‐tailed with a significance threshold of *p* < 0.05. Analyses were conducted using Statistical Analysis System (SAS) version 9.4 (SAS Institute, Cary, New Carolina, USA).

### 2.5 Ethics Statement

Patients were recruited using opt‐out consent. Ethics approval was obtained from the Monash University Human Research Ethics Committee (MUHREC ID 30372).

## 3 Results

### 3.1 Participants

A total of 14,559 patients with NSCLC were identified, of whom 4511 (30.9%) resided in regional areas at the time of diagnosis. Smoking status differed across geographic locations with 2848/9304 (31%) current smokers in metropolitan areas, 366/1083 (34%) in regional areas and 1148/3161 (36%) in rural areas (*p* < 0.001). Regional and rural patients were more often Australian‐born (metropolitan, 4919/9304 [53%] vs. regional, 873/1083 [81%] vs. rural, 2608/3161 [83%]; *p* < 0.001) (Table 1).

**TABLE 1:** Baseline demographic characteristics of patients with non‐small cell lung cancer, stratified by geographic location.

Characteristic	Metropolitan (MM1)^a^	Regional (MM2)^a^	Rural and remote (MM3–7)^a^	*p*
Total number of patients (% of total cohort)	9304/13,548 (69%)	1083/13,548 (8%)	3161/13,548 (23%)	
Median age, years (IQR)	70.0 (63–77)	70.0 (62–76)	70.0 (63–76)	< 0.001
Sex				
Female	4162 (45%)	473 (44%)	1371 (43%)	0.37
Male	5142 (55%)	610 (56%)	1790 (57%)	
Year of diagnosis				
2011–2015	1847 (20%)	151 (14%)	565 (18%)	< 0.001
2016–2019	4284 (46%)	521 (48%)	1534 (48%)	
2020–2022	3173 (34%)	411 (38%)	1062 (34%)	
Smoking status				
Current smoker	2848 (31%)	366 (34%)	1148 (36%)	< 0.001
Former smoker	4707 (51%)	572 (53%)	1674 (53%)	
Never smoked	1451 (16%)	111 (10%)	251 (8%)	
Not known	298 (3%)	34 (3%)	88 (3%)	
Continent of birth				
Australia	4919 (53%)	873 (81%)	2608 (83%)	< 0.001
Other	4385 (47%)	210 (19%)	553 (17%)	< 0.001
Indigenous status				
Indigenous	61 (1%)	19 (2%)	52 (2%)	< 0.001
Non‐Indigenous	9243 (99%)	1064 (98%)	3109 (98%)	
ECOG status^b^				
ECOG 0	2303 (25%)	236 (22%)	697 (22%)	< 0.001
ECOG 1	2579 (28%)	362 (33%)	978 (31%)	
ECOG 2	848 (9%)	93 (9%)	320 (10%)	
ECOG 3	377 (4%)	48 (4%)	123 (4%)	
ECOG 4	58 (1%)	5 (1%)	13 (1%)	
Not known	3139 (33%)	339 (31%)	1030 (33%)	
Socio‐economic status				
Suburb‐level IRSAD^c^ decile, median (IQR)	6.0 (3.0–9.0)	4.0 (2.0–7.0)	3.0 (2.0–5.0)	< 0.001
Driving distance to treating hospital, hours				
< 1	9112 (98%)	720 (67%)	659 (21%)	< 0.001
1–3	179 (2%)	184 (17%)	1531 (48%)	
> 3	13 (0.1%)	179 (17%)	971 (31%)	
Location of treating hospital^a^				
MM1	4278 (46%)	178 (16%)	446 (14%)	< 0.001
MM2	9 (0.1)	321 (30%)	720 (23%)	
MM3	5 (0.1)	0	221 (7%)	
MM4	1 (0.1)	1 (0.1%)	56 (2%)	
MM5	1 (0.1)	0	1 (0.1)	
Did not require systemic therapy or have treatment	5010 (54%)	583 (54%)	1717 (54%)	
Patient clinical stage				
I	1357 (15%)	200 (18%)	577 (18%)	< 0.001
II	696 (7%)	118 (11%)	284 (9%)	
III	1386 (15%)	191 (18%)	548 (17%)	
IV	3994 (43%)	411 (38%)	1215 (38%)	
Not known	1871 (20%)	163 (15%)	537 (17%)	
Histology^d^				
Adenocarcinoma	5892 (63%)	575 (53%)	1761 (56%)	< 0.001
Squamous	1890 (20%)	253 (23%)	716 (23%)	
Other	1522 (16%)	255 (24%)	684 (22%)	
Treatment				
Surgery	2820 (30%)	353 (33%)	982 (31%)	0.14
Radiotherapy	3120 (34%)	369 (34%)	1081 (34%)	
Systemic anti‐cancer therapy	3364 (36%)	361 (33%)	1098 (35%)	
Other medical conditions, median (IQR)	1.0 (0.0–1.0)	1.0 (0.0–1.0)	1.0 (0.0–1.0)	0.19
Referral to palliative care	2888 (31%)	365 (34%)	880 (28%)	< 0.001
Clinical trial enrolment	260 (3%)	50 (5%)	75 (2%)	

Abbreviations: ECOG, Eastern Cooperative Oncology Group; Indigenous status, Aboriginal and/or Torres Strait Islander; IQR, interquartile range; IRSAD, Index of Relative Socio‐Economic Advantage and Disadvantage; NOS, not otherwise specified histology.

^a^ Geographic location is classified according to the Modified Monash Model (MMM), with Modified Monash (MM) category 1 (MM1) representing metropolitan areas and MM2–MM7 representing progressively more rural and remote areas.

^b^ ECOG 0–1 indicates patients who are fully active or restricted in physically strenuous activity but ambulatory. Missing data are excluded from some percentage calculations; categories may not add up to total *N* due to missing values.

^c^ IRSAD quintiles range from 1 (most disadvantaged) to 5 (least disadvantaged), based on residential postcode.

^d^ Other histologies include mixed adenosquamous, large cell carcinoma, not otherwise specified.

### 3.2 Descriptive Data

#### 3.2.1 Access to Treatment Centres

There were differences in accessibility to treatment and healthcare infrastructure between metropolitan and regional/rural patients. Patients in regional and rural areas had greater geographical barriers to treatment, with longer driving distances to treatment centres. The mean driving time to the nearest treatment institution was 0.4 h (standard deviation [SD], 0.8) for metropolitan patients, 1.9 h (SD, 4.5) for regional patients and 2.8 h (SD, 2.6) for rural and remote patients. Regional and rural patients travelled longer distances for treatment, with more patients travelling > 3 h (metropolitan, 13/9304 [0.1%] vs. regional, 179 [16.5%] vs. rural, 971/3161 [31%]; *p* < 0.001) (Table 1).

#### 3.2.2 Socio‐Economic Differences

Regional and rural patients had lower IRSAD scores, indicating greater socio‐economic disadvantage. The median IRSAD decile was 6.0 (interquartile range [IQR], 3.0–9.0) for metropolitan patients, 4.0 (IQR, 2.0–7.0) for regional patients and 3.0 (IQR, 2.0–5.0) for rural patients (*p* < 0.001), indicating a strong association between geographic location and socio‐economic disadvantage (Table 1).

#### 3.2.3 Treatment Location Patterns Among Regional and Rural Patients

A substantial proportion of patients from regional and rural areas received systemic anti‐cancer therapy at metropolitan institutions, reflecting overlapping treatment pathways across geographic regions. Specifically, 178/1083 (16.4%) regional patients and 446/3161 (14.1%) rural and remote patients accessed treatment in metropolitan centres. These patterns suggest multifactorial influences on treatment location, potentially involving availability of services, referral practices and individual patient preference or clinician decision‐making.

### 3.3 Outcome Data

#### 3.3.1 Survival Analysis

**FIGURE 1:**
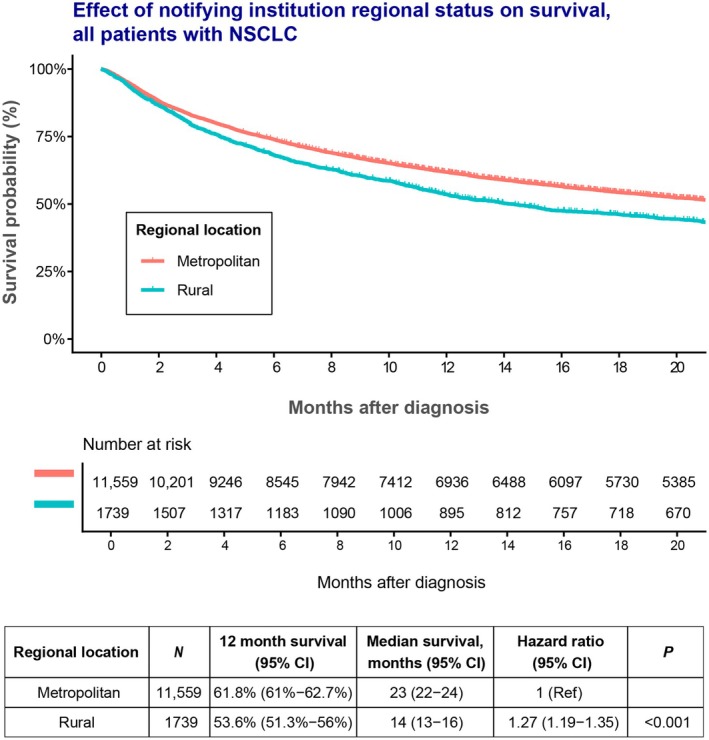
Kaplan–Meier survival curves showing overall survival for patients with non‐small cell lung cancer (NSCLC), stratified by geographic location of the notifying institution.^a^ CI, confidence interval. ^a^The inset table presents 12‐month survival, median survival and hazard ratios from unadjusted Cox regression analysis. For the purpose of the survival analysis, regional, rural and remote areas were combined into a single group and referred to as ‘rural’.

For the purpose of the survival analysis, regional, rural and remote areas were combined into a single group and referred to as ‘rural’. Overall survival outcomes were poorer for patients treated in rural institutions compared with those treated in metropolitan institutions. The unadjusted HR for mortality among rurally treated patients was 1.27 (95% CI, 1.19–1.35), suggesting a 27% higher risk of mortality compared with metropolitan patients (Figure 1). After adjusting for key prognostic variables including age, ECOG performance status, cancer stage, smoking status, year of diagnosis and comorbidities, the association persisted, with a multivariate‐adjusted HR of 1.11 (95% CI, 1.04–1.18). These findings highlight the persistent survival differences between metropolitan and regional patients with lung cancer despite accounting for known clinical and demographic confounders.

**FIGURE 2:**
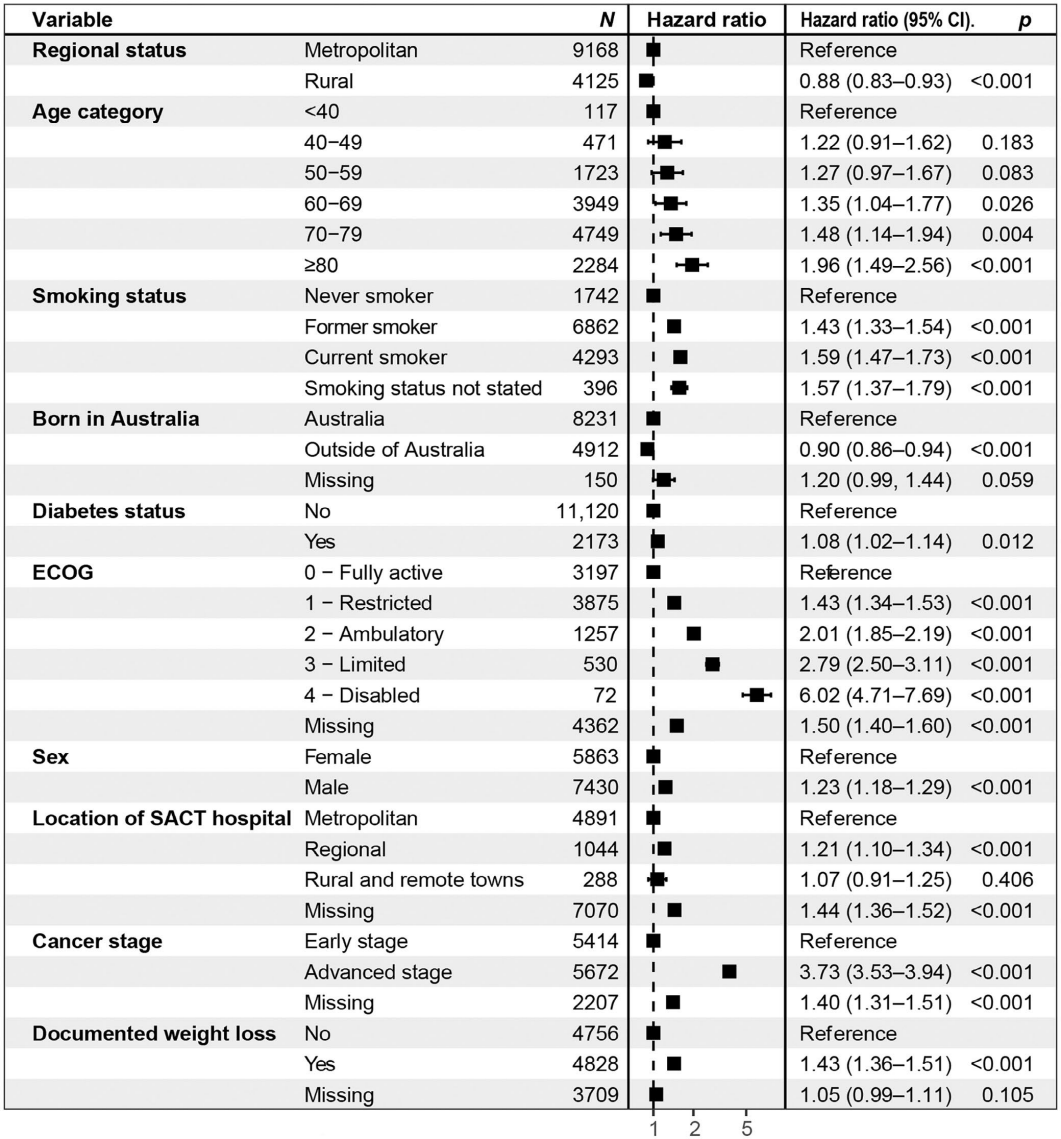
Multivariate Cox regression forest plot of prognostic factors for overall survival in non‐small cell lung cancer. CI, confidence interval; ECOG, Eastern Cooperative Oncology Group; SACT, systemic anti‐cancer therapy.

Other variables tested showed an expected correlation with survival outcomes such as increasing age, poorer performance status, current and ex‐smoking status and advancing clinical stage, all of which were associated with greater mortality risk (Figure 2). Increasing age was associated with reduced overall survival per year (adjusted HR [aHR], 1.22; 95% CI, 0.91–1.62), and male patients had a higher risk of mortality than female patients (aHR, 1.18; 95% CI, 1.13–1.24). Performance status was a strong predictor of survival, with poorer ECOG scores correlating with greater mortality risk. Compared with ECOG 0, patients with ECOG 1 had a 43% higher risk of mortality (HR, 1.43; 95% CI, 1.34–1.53), which increased progressively with ECOG 2 (HR, 2.01; 95% CI, 1.85–2.19), ECOG 3 (HR, 2.79; 95% CI, 2.50–3.11) and ECOG 4 (HR, 6.02; 95% CI, 4.71–7.69).

Smoking status also played a critical role in survival outcomes. Compared with never‐smokers, ex‐smokers had a 42% higher mortality risk (HR, 1.43; 95% CI, 1.33–1.54) and current smokers had an even greater risk (HR, 1.59; 95% CI, 1.47–1.73). Similarly, cancer stage at diagnosis was strongly associated with prognosis. Compared with patients diagnosed at stage I, those diagnosed at stage II had an 87% increased risk of mortality (HR, 1.87; 95% CI, 1.66–2.11), which more than tripled at stage III (HR, 3.17; 95% CI, 2.87–3.5) and increased nearly sevenfold at stage IV (HR, 6.74; 95% CI, 6.16–7.38).

Lastly, comorbidities had a smaller yet statistically significant impact on survival, with each additional comorbidity slightly increasing mortality risk (HR, 1.04; 95% CI, 1.01–1.07). These findings highlight the critical influence of demographic and clinical characteristics on overall survival in patients with NSCLC and emphasise the differences faced by those treated in regional and rural settings.

#### 3.3.2 Sensitivity Analysis

**FIGURE 3:**
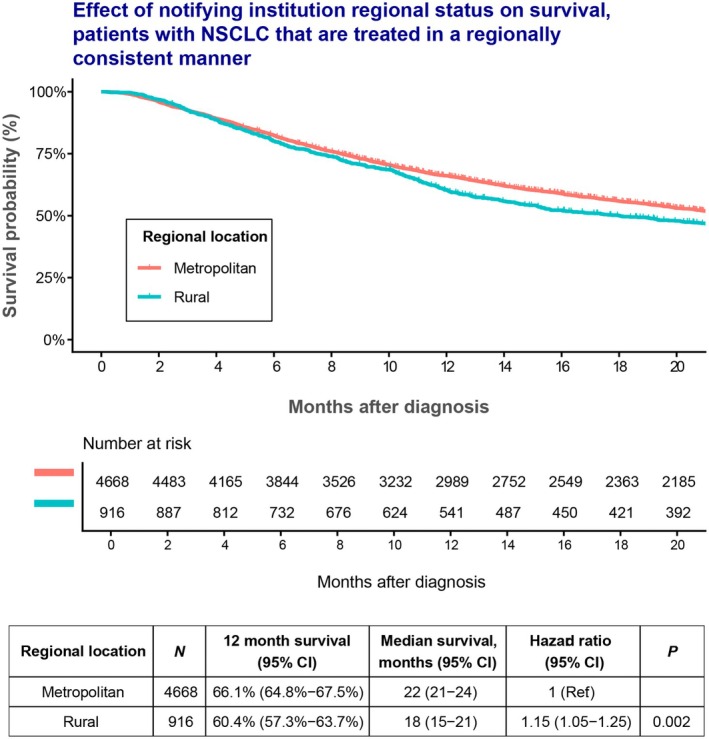
Kaplan–Meier survival curves excluding regional/rural patients with non‐small cell lung cancer (NSCLC) who received treatment in metropolitan centres, assessing the independent effect of residence on survival.^a^ CI, confidence interval. ^a^For the purpose of the survival analysis, regional, rural and remote areas were combined into a single group and referred to as ‘rural’.

A notable proportion of regional (178/1083, 16.4%) and rural and remote (446/3161, 14.1%) patients received their systemic anti‐cancer therapy at metropolitan institutions, which could potentially confound survival comparisons by treatment location. To account for this, we excluded these patients from the primary survival analysis. Even after their removal, the survival difference persisted: median survival was 22 months (IQR, 20.0–23.0 months) in the metropolitan group compared with 18 months (IQR, 17.0–20.0 months) in the combined rural and remote group (HR, 1.15; 95% CI, 1.05–1.25) (Figure 3).

This finding was further supported by a propensity score–matched analysis, which controlled for key variables including age, sex, ECOG performance status, smoking status, clinical stage, number of comorbidities and year of diagnosis. The survival difference remained statistically significant (HR, 1.09; 95% CI, 1.01–1.17).

## 4 Discussion

This population‐based study shows significant survival disparities between metropolitan, regional and rural patients with NSCLC. Our findings demonstrate that regional and rural patients face notable barriers, including longer travel times to treatment centres, lower socio‐economic status and a higher prevalence of smoking. These factors likely contribute to the observed 27% increased unadjusted mortality risk in regional patients, which remained significant even after adjusting for key prognostic variables (HR, 1.11; 95% CI, 1.04–1.18). The interplay of patient‐level characteristics (such as smoking), socio‐economic disadvantage and geographic barriers to accessing care are likely key contributors to the survival disparity observed [15].

One of the most striking findings was the overlap in treatment locations, with a significant proportion of regional and rural patients receiving care at metropolitan institutions. About 16.4% of regional patients and 14.1% of rural patients received their systemic anti‐cancer therapy at a metropolitan centre. This pattern suggests a complex interplay of factors influencing treatment location, potentially including healthcare system logistics, availability of specialised services, referral pathways and patient or clinician perceptions of care quality [16, 17]. It also highlights possible gaps in the accessibility or capacity of regional cancer services, which may be compounded by geographic and socio‐economic barriers.

Another important finding in our analysis was the pronounced socio‐economic disadvantage observed in regional compared with metropolitan patients. Patients from regional and rural areas had significantly lower IRSAD scores, indicating higher levels of economic and educational disadvantage. The median IRSAD decile was 4.0 (IQR, 2.0–7.0) for regional patients and 3.0 (IQR, 2.0–5.0) for rural patients, compared with 6.0 (IQR, 3.0–9.0) for those residing in metropolitan areas. Socio‐economic disadvantage is linked to poorer health outcomes by influencing health‐seeking behaviours, risk exposure, lifestyle factors, insurance status and access to timely care, contributing to delayed diagnoses, reduced treatment adherence and worse prognosis. Variability in the uptake and access to services such as smoking cessation programs, allied health support and preventive care may also contribute to the observed differences in outcomes across geographic groups [18, 19, 20, 21, 22].

In addition, clinical characteristics such as smoking status and performance status played a critical role in survival outcomes. The larger proportion of current and ex‐smokers in regional and rural cohorts aligns with the greater mortality risk associated with smoking (HR, 1.59 [95% CI, 1.47–1.73] for current smokers vs. 1.43 [95% CI, 1.33–1.54] for ex‐smokers). Similarly, poorer performance status was strongly associated with higher mortality risk, with ECOG scores of 2 or greater predicting a significantly worse prognosis. These findings emphasise the need for targeted interventions to address modifiable risk factors, such as smoking cessation, physical deconditioning and comorbidity management, while also improving supportive care for high‐risk patients and ensuring equitable access to treatment services across all geographic areas [23].

A recent publication from Queensland identified the percentage of people from rural/remote areas who received their first treatment within 30 days of diagnosis fell from 49% in 2012–2016 to 44% between 2017 and 2021. This decrease was only observed for people with NSCLC from rural/remote areas who were treated at public hospitals, among whom first treatment within 30 days of diagnosis dropped from 48% to 36%, whereas this indicator remained stable for those treated at private hospitals [24].

Given these findings, strengthening regional healthcare infrastructure should be a priority, particularly as Australia moves towards the implementation of lung cancer screening programs. Ensuring that regional and rural patients have timely access to treatment within their region, diagnostic services, multidisciplinary care, preventive health services and advanced treatments will be essential in reducing survival disparities and improving long‐term outcomes for all patients with NSCLC.

Strengths of this study include the large, population‐based cohort of patients, enabling robust and generalisable insights. The use of risk‐adjusted and standardised data collection, facilitated by the registry infrastructure ensures data consistency and completeness across multiple sites. Selection bias was minimised by the inclusion of all consecutive patients at each participating institution.

In addition, the dataset incorporated detailed clinical, demographic and geographic variables, allowing for a nuanced evaluation of prognostic factors and treatment access. The study also leveraged real‐world data from both metropolitan and regional settings, enhancing the relevance of findings to clinical practice and health system planning.

### 4.1 Limitations

Although these findings provide valuable insight into regional disparities, several limitations must be acknowledged. The data used in this study were obtained from a cancer registry and there is a possibility that regional patients may be underrepresented due to potentially reduced resources in regional institutions for data collection and registry reporting. In addition, the overlap in treatment locations complicates direct comparisons between metropolitan and regional outcomes, as some regional patients receive care in metropolitan settings.

Findings may not be fully generalisable to other Australian states or territories, as health service organisation, population distribution, geographic remoteness and availability of specialist cancer services differ across jurisdictions. Future research should focus on refining data collection methods and exploring patient decision‐making processes to better understand regional healthcare access patterns.

Unmeasured confounding may bias results due to the lack of data on important factors such as patient frailty, individual treatment preferences, social support and caregiver availability variables that are often not captured in registry‐based datasets. A second limitation is the retrospective study design. Furthermore, the use of MMM administrative classifications for treatment institution locations may not fully reflect differences in care quality or the specific logistical and workforce challenges faced by individual regional healthcare providers. To account for potential unmeasured confounding, a sensitivity analysis was conducted, which demonstrated consistent findings across analytical approaches. This strengthens the validity of the results and suggests that the observed associations are robust despite the limitations of unmeasured variables.

## 5 Conclusions

Our study highlights significant survival differences between regional, rural and remote and metropolitan patients with NSCLC in Victoria. The findings underscore the importance of addressing geographic disparities in access to care, improving early detection strategies and enhancing healthcare infrastructure in rural areas. Further research, including qualitative studies exploring patient and provider experiences, could provide deeper insights into the factors influencing survival disparities. A deep dive into understanding the finer details of the hospital and infrastructure differences between metropolitan and regional institutions will be important. Increasing regional representation in cancer and health registries should be a priority.

## Author Contributions

Evangeline Samuel: Conceptualisation, methodology, formal analysis, data curation, writing (original draft), visualisation, project administration. Eldho Paul: Formal analysis, methodology, writing (review and editing). Mike Lloyd, Sanuki Tissera, Jessie Zeng: Data curation, investigation, writing (review and editing). Craig Underhill, Sagun Parakh, Phillip Parente, Inger Olesen, Javier Torres, Katharine See, David Langton, Tom John, Gavin M. Wright, Matthew Conron, James Bartlett, Nicola Atkin: Investigation, resources, writing (review and editing). Nik Zeps, Susan V. Harden: Methodology, supervision, writing (review and editing). Wasek Faisal: Investigation, writing (review and editing). John Zalcberg: Conceptualisation, supervision, writing (review and editing). Rob G. Stirling: Conceptualisation, methodology, supervision, writing (review and editing). All authors reviewed and approved the final version of the manuscript.

## Funding

The authors have nothing to report.

## Conflicts of Interest

The authors declare no conflicts of interest.

## Supporting information


**Table S1:** STROBE checklist.

## Data Availability

The data supporting the findings of this study are available from the corresponding author upon request.
