# Supplementary material for: Uneven Ground: Survival Differences Among Victorian Lung Cancer Patients by Location of Residence (2011–2023): A Retrospective Cohort Study
Source: Med J Aust. 2026 Aug 18;224(8):e70268. doi: 10.5694/mja2.70268 (PMC13484183; doi:10.5694/mja2.70268)
Supplement: Supplementary file 1 [file MJA2-224-0-s001.pdf]

MJA
The Medical Journal of Australia

Supporting Information

Supplementary material

This appendix was part of the submitted manuscript and has been peer reviewed.
It is posted as supplied by the authors.

Appendix to: E. Samuel, E. Paul, M. Lloyd, et al. Uneven Ground: Survival Differences Among Victorian Lung Cancer Patients by Location of Residence (2011–2023): A Retrospective Cohort Study. *Med J Aust* 2026; doi: 10.5694/mja2.70268.

Supplementary Table S1.STROBE checklist.

Section/Item	Recommendation	Page No.	Notes
Title	Indicate study design	Title	“Retrospective cohort study” included
Abstract	Structured abstract	1	Structured abstract included
Introduction	Background/rationale and specify objectives	Page 2	Explained scientific background and specified objectives which is it compare survival by residence
Methods			Pages 5-7
Setting	Describe locations, dates of recruitment, and follow-up	Methods page 5	Victoria, VLCR data, dates of recruitment and follow up included
Participants	Eligibility criteria	Methods page 5	Inclusion/exclusion described
Variables	Define exposures/outcomes	Methods page 5	All data variables described
Bias	Address bias	Methods page 5	Adjusted Cox models- described in page 6
Study size	Explain sample size	Methods page 5	Registry-based cohort
Statistical methods	Describe analysis	Methods page 6-7	Detailed statistical analyses described
Results		Results pages 7	
Participants		Pages 7	Participants described
Descriptive data	Baseline characteristics	Pages 7-8	Table 1
Outcome data	Report outcomes	Pages 7-8	Outcome details explained
Main results	Adjusted estimates	Pages 8-9	Figures 1,2,3
Discussion	Key results	Discussion pages 10	Discussion explained
Limitations	Discuss limitations	Limitations pages 12	Limitations explained
Interpretation	Overall interpretation	Discussion pages 10	
Funding	Funding sources	End	
